# Epidemiology of MRSA at the University of Geneva Hospitals

**DOI:** 10.1186/1753-6561-5-S6-P5

**Published:** 2011-06-29

**Authors:** CM Fankhauser, J Schrenzel, D Pittet, S Harbarth

**Affiliations:** 1 Infection Control Programme, Geneva, Switzerland; 2 Genomics Research Laboratory, University of Geneva Hospitals, Geneva, Switzerland

## Introduction / objectives

Curbing MRSA has been a challenge for our institution, a hospital with hyperendemic MRSA since 1999.

## Methods

An observational cohort study on MRSA trends was prospectively carried out from 1993 onwards. Infection control measures were initiated and intensified at various time points, including patient screening, surveillance, contact isolation, a computerized alert system and hospital-wide promotion campaign of hand hygiene. In 2003, the Vigigerm® program was introduced, 2006 a second promotion campaign of hand hygiene took place. The MRSA burden had been recorded by indicators as the proportion of MRSA among *S. aureus* in blood cultures (expressed as percentage); validated on site surveillance data of MRSA infected or colonized patients expressed as incidence rates and MRSA incidence-density, expressed as acquired MRSA cases per 1000 hospital days.

## Results

Two distinct periods could be observed. Increasing rates of newly MRSA-infected or -colonized patients were observed from 1989-1994 (from 0.05 to 0.6 cases per 100 admissions); by 1997 it has decreased to 0.24. Since 2000, there was a significant increase in the MRSA burden, recorded by all indicators. This coincided with the introduction of a highly epidemic clone (ST228 South German). From 2000-2006 the incidence rate grew from 1.36 to 2.00 new cases per 100 admissions; with a plateau between 2006 and 2008. It began to decrease by 2008 (from 1.70 to 1.12 new cases per 100 admissions). The attack rate followed the same pattern (decline from 1.36 in 2007 to 0.70 acquired MRSA/1000 per hospital days in 2010). Since 2000, the proportion of MRSA among *S. aureus* in blood cultures stayed around 30%, with a marked decrease in 2010 to 23%.

## Conclusion

An ongoing intensive MRSA control program is necessary to contain endemic MRSA rates.

## Disclosure of interest

None declared.

